# Supplementary figures and images for: ERSAtool: A User‐Friendly R/Shiny Comprehensive Transcriptomic Analysis Interface Suitable for Education
Source: Genes Cells. 2025 Aug 18;30(5):e70044. doi: 10.1111/gtc.70044 (PMC12360868; doi:10.1111/gtc.70044)

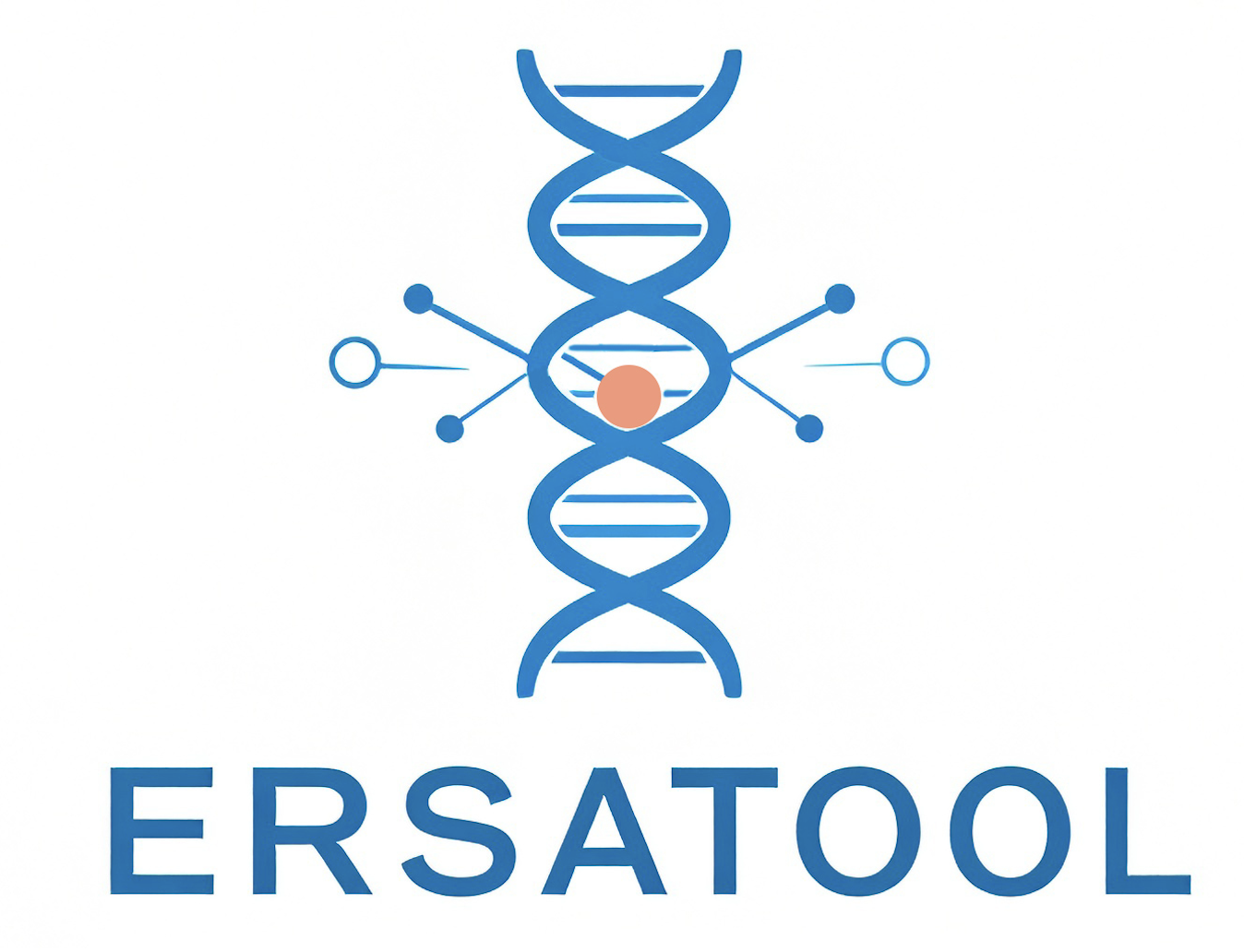

Supplement: Supplementary file 2 — Supporting Information 2. ERSAtool app (ERSAtool‐main.zip). [file GTC-30-0-s004.zip › ERSAtool-main/www/ERSA.png]

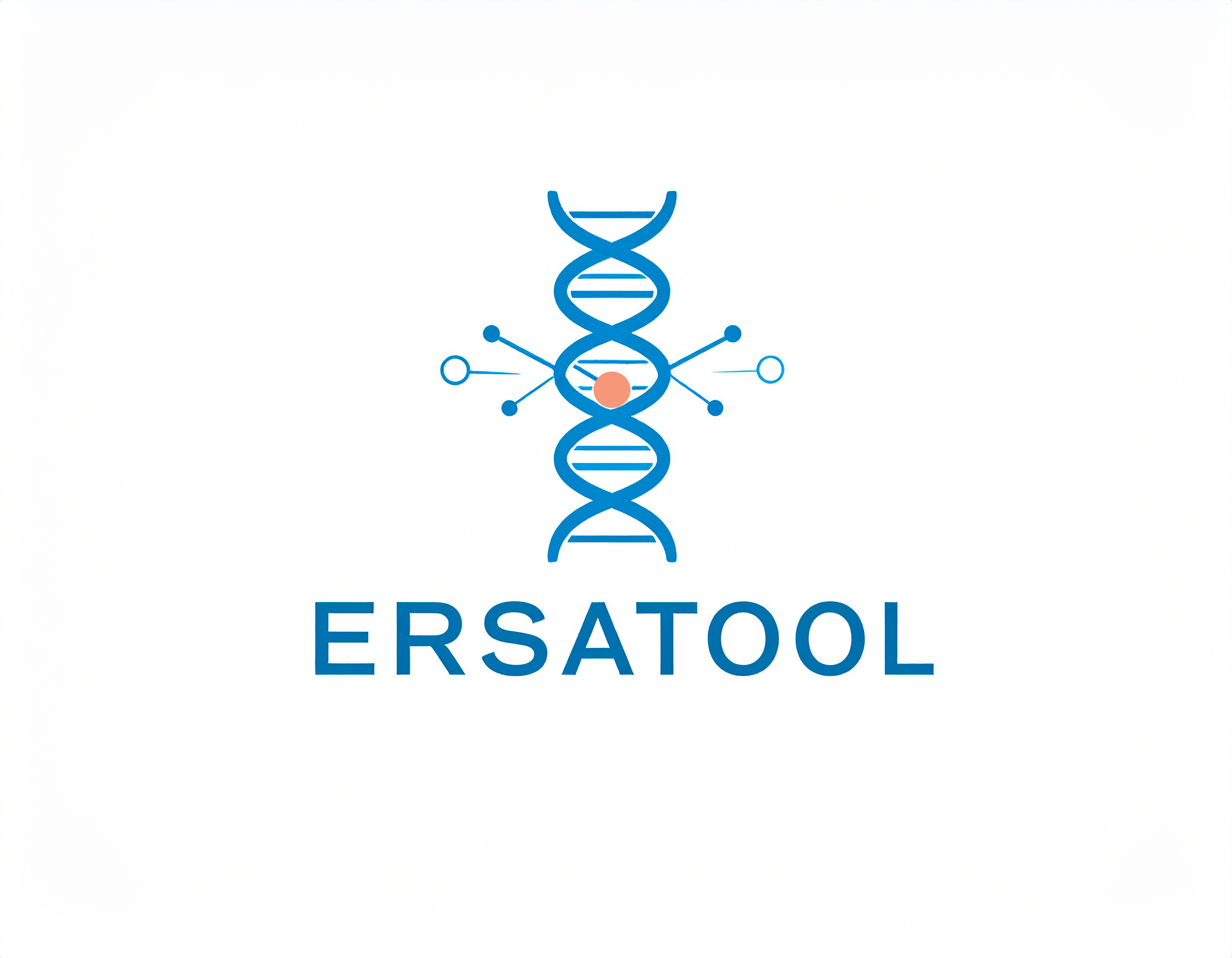

Supplement: Supplementary file 2 — Supporting Information 2. ERSAtool app (ERSAtool‐main.zip). [file GTC-30-0-s004.zip › ERSAtool-main/www/ERSA_logo6.jpg]

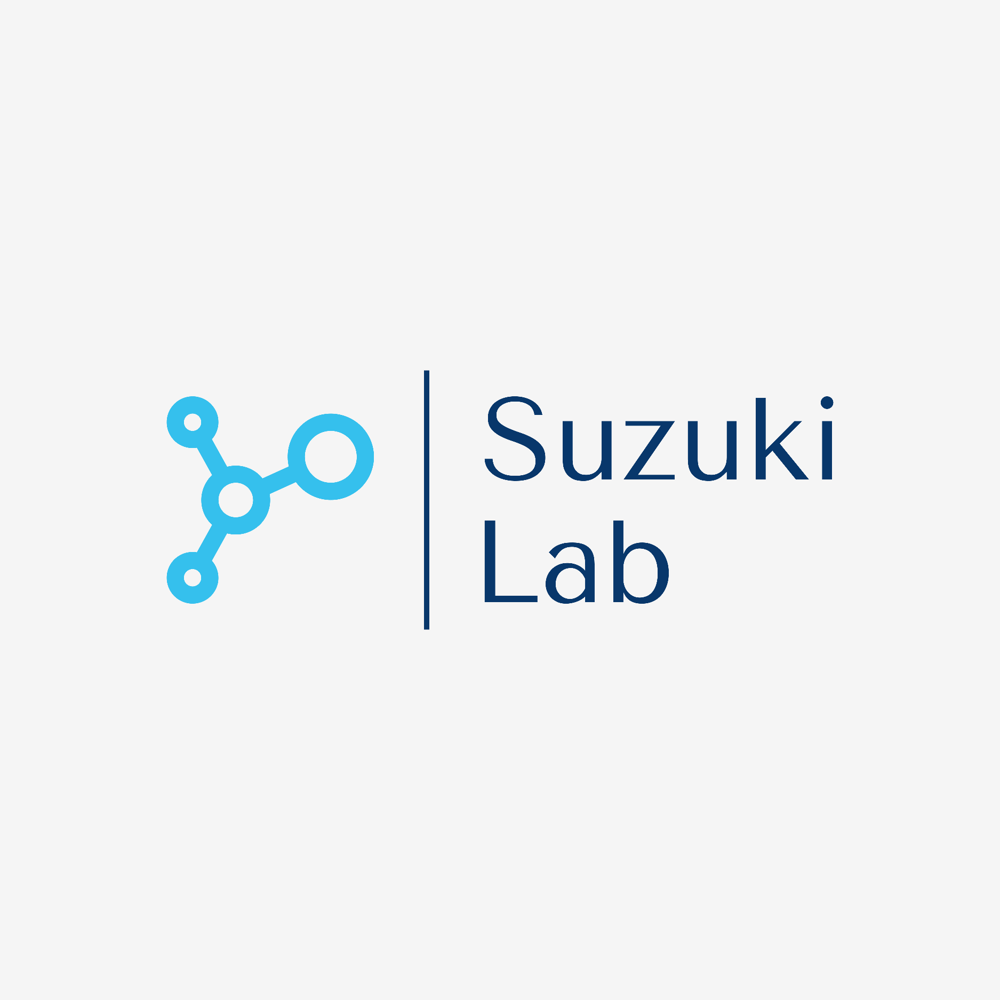

Supplement: Supplementary file 2 — Supporting Information 2. ERSAtool app (ERSAtool‐main.zip). [file GTC-30-0-s004.zip › ERSAtool-main/www/Suzukilab_logo.png]

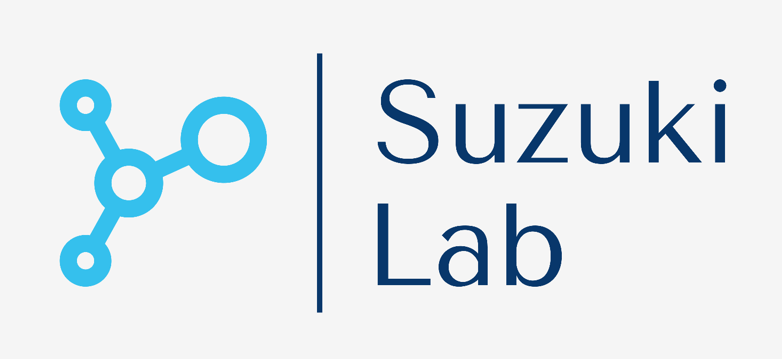

Supplement: Supplementary file 2 — Supporting Information 2. ERSAtool app (ERSAtool‐main.zip). [file GTC-30-0-s004.zip › ERSAtool-main/www/Suzukilab_logo_cropped.png]
